# Solitary pulmonary mass in a patient with a history of lymphoma: a case report

**DOI:** 10.1186/1752-1947-7-113

**Published:** 2013-04-25

**Authors:** Ying Yuan, Hong Shen, Hanguang Hu, Xiaoxian Ye, Xian Zhong

**Affiliations:** 1 Department of Medical Oncology, the Second Affiliated Hospital of Zhejiang University College of Medicine, Hangzhou, China; 2 Binjiang branch of the Second Affiliated Hospital of Zhejiang University College of Medicine, Hangzhou, China; 3 Hangzhou Binjiang Hospital, Hangzhou, China

## Abstract

**Introduction:**

With the progress made in treatments, the survival rate for patients with malignant lymphoma in the last 30 years has significantly improved. However, the risk of experiencing a second primary malignancy or other disease has increased significantly.

**Case presentation:**

A 44-year-old Mongolian man with a large mass in his right lower abdomen was admitted to our hospital 15 years previously. The mass was removed, and confirmed via pathological examination to be a malignant B-cell lymphoma in the appendix and distal small bowel. Post-operative chemotherapy with standard cyclophosphamide, hydroxydaunomycin, vincristine (Oncovin®) and prednisolone regimen was given for six cycles. No obvious recurrence was detected over the following 12 years. Subsequently, a mass in the right lung was found on a regular X-ray follow-up; our patient did not report chills, fever or cough. Chest computed tomography and positron emission tomography scans confirmed the mass. A primary lung carcinoma was considered to be the most likely diagnosis. However, after an exploratory thoracotomy and right upper lobectomy was performed a pathological examination of tissue samples demonstrated a lung cryptococcal granuloma, with positive staining for periodic acid Schiff and periodic acid-silver metheramine.

**Conclusions:**

Compared to the normal population, second primary malignancy (in particular leukaemia and lung cancer) in patients with malignant lymphoma during their long-term survival has been seen occasionally. However, other diagnoses should also be considered such as pulmonary cryptococcosis. Other than computed-tomography-guided needle biopsy, surgery for some patients is a much more appropriate choice, which could also help attain correct diagnosis and treatment.

## Introduction

With the progress made in treatments, in the last 30 years the survival rate for patients with malignant lymphoma has significantly improved. Use of the targeted drug rituximab in CD20(+) B-cell lymphoma has greatly increased the efficiency of treatment and five-year survival rate. However, compared to the normal population, the risk of suffering a second primary malignancy, in particular leukemia and lung cancer, has increased significantly in patients with malignant lymphoma during their long-term survival after treatment [1-3]. Usually, the first diagnosis in these patients with a mass found during regular follow-up is a tumor. However, on occasion this could be the wrong conclusion.

## Case presentation

**Figure 1:**
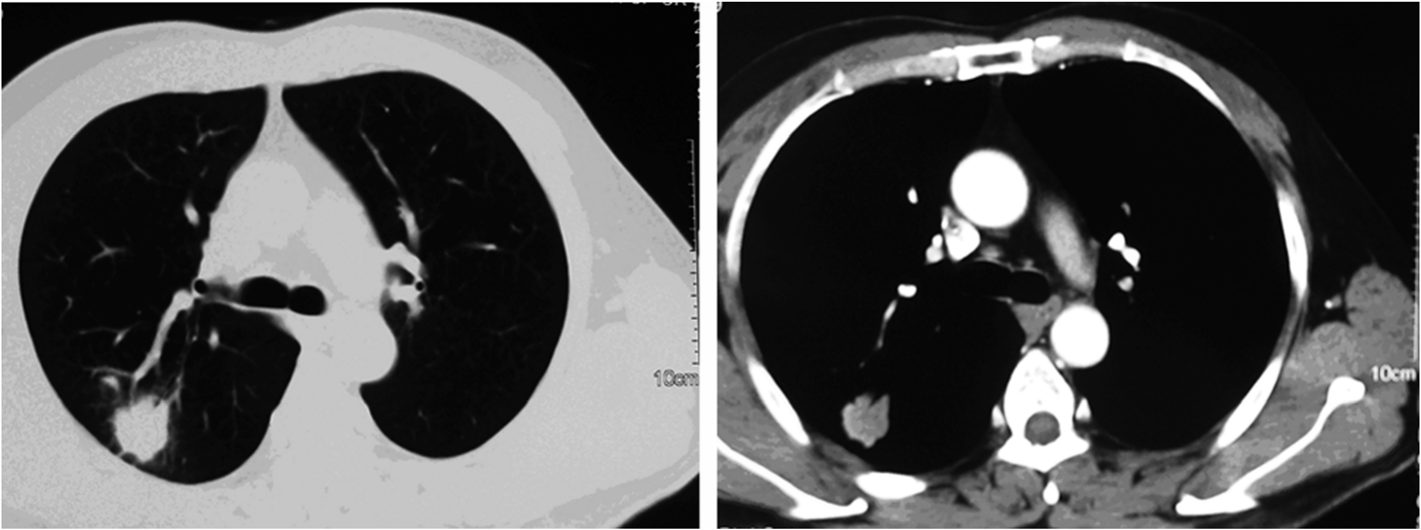
Chest computed tomography scan showing a mass with signs of lobulation and burr, sized 2.4×2.0cm, in the dorsal section of the right lung.

**Figure 2:**
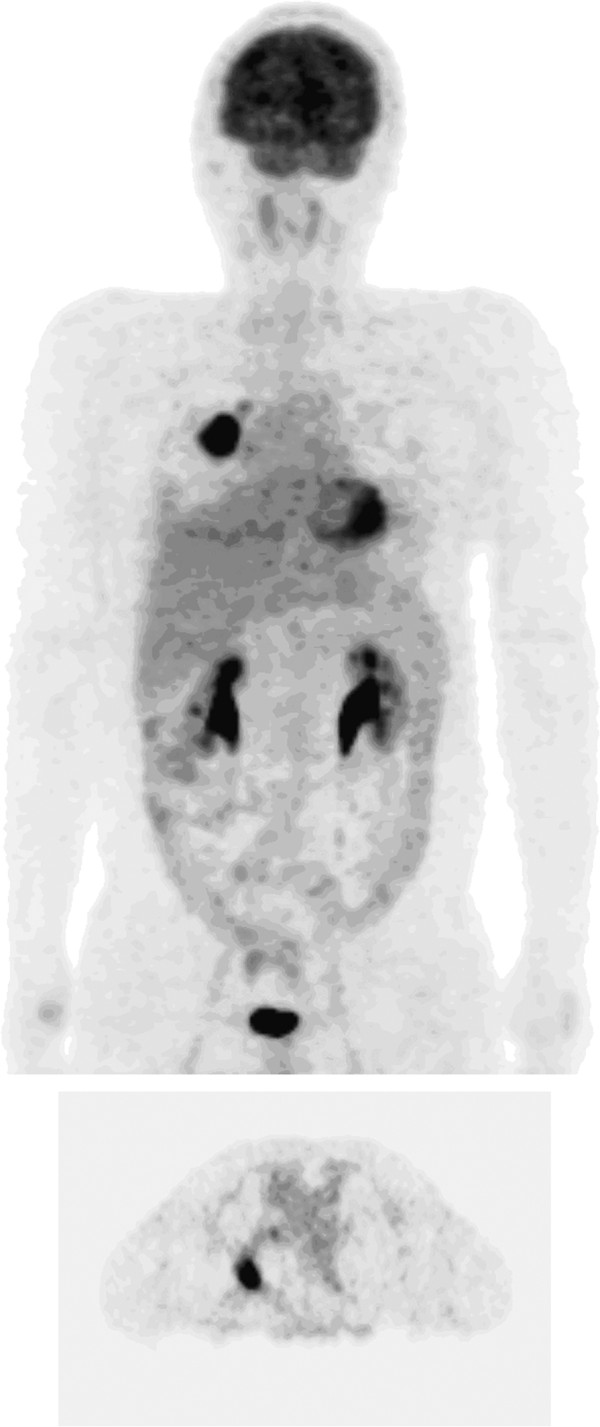
An 18F-fluorodeoxyglucose (FDG) positron electron tomography (PET) scan showing lesions with obviously increased standardized uptake values (SUVs) in the lower lobe and hilum of the right lung.

A 44-year-old Mongolian man with a large mass in his right lower abdomen presented to our hospital 15 years ago. After completion of relevant physical and laboratory examinations, abdominal laparotomy was performed under general anesthesia, and the mass was removed. Pathological examination and immunohistochemical staining of tissue samples showed a malignant B-cell lymphoma in the appendix and small bowel. Post-operative chemotherapy with a standard cyclophosphamide, hydroxydaunomycin, vincristine (Oncovin®) and prednisolone (CHOP) regimen was given for six cycles. No obvious recurrence was detected during regular follow-up appointments over the following 12 years. Then, three years ago, a mass in the right lung was detected during a regular X-ray follow-up. Our patient reported no chills, fever or cough. The results of a physical examination were unremarkable. A chest computed tomography (CT) scan showed a mass with lobulation and burr in the dorsal section of the right lung, sized 2.4×2.0cm (Figure 1). An 18 F-fluorodeoxyglucose (FDG) positron emission tomography (PET) scan showed lesions with obviously increased standardized uptake values (SUVs) in the lower lobe and hilum of the right lung. The maximum SUVs of the lesions on the initial scans were 17.8 and 4.1, respectively, while the maximum values on later scans were elevated to 22.0 and 4.3, respectively (Figure 2). A bone scintigraphy scan, brain magnetic resonance (MRI) and ultrasound examination of the upper abdomen all showed no abnormal signs. The results of an anti-human immunodeficiency virus (HIV) antibody test were negative. A tuberculin test was also negative.

**Figure 3:**
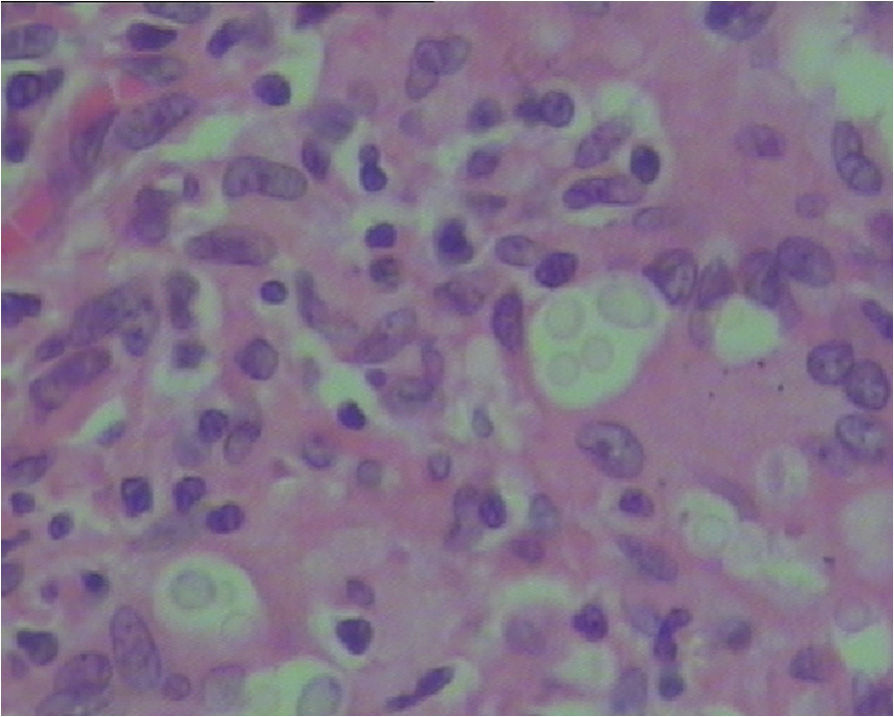
A pathological examination demonstrated lung cryptococcal granuloma (×400).

A multidisciplinary discussion of our patient’s case was carried out. Primary lung carcinoma was considered to be the most likely diagnosis. Our patient refused to undergo a CT-guided needle biopsy and asked for exploratory thoracotomy instead. Considering our patient’s wishes, a right exploratory thoracotomy and right upper lobectomy was performed under general anesthesia. Pathological examination of samples showed lung cryptococcal granuloma, with positive staining with periodic acid Schiff (PAS) and periodic acid-silver metheramine (PASM). No acid-fast bacilli were found. All the lymph nodes including six of the bronchial lymph nodes, two of group 2, two of group 3, five of group 4, five of group 7 and one of group 8 showed chronic inflammation (Figure 3). Our patient was treated with fluconazole tablets post-operatively (0.2g once a day orally) for anti-fungal treatment for three months. Our patient was followed up by monthly review; no recurrence had been found to date, more than 18 months after he underwent chest surgery.

## Conclusions

Diagnosis of pulmonary cryptococcosis usually depends on the etiology of infected tissue cultures or pathology findings. Surgery, percutaneous needle biopsy, bronchoscopy brushing or biopsy, bronchoalveolar lavage fluid culture, smear or culture of cerebrospinal fluid and lymph node biopsy are all effective methods for diagnosis. Combined with the results of PET and lung CT scans, our patient was suspected to have a second tumor: primary lung cancer. However, pathological examination verified the primary pulmonary cryptococcosis, caused by infection of *Cryptococcus neoformans*. Our patient denied an exposure history of bird secretions, dust and pollen. Previous studies have reported that T-cell-mediated immune dysfunction often occurs in patients with cancer after chemotherapy, who are susceptible to pulmonary cryptococcosis [4,5]. We believe that surgery for such patients is a more appropriate choice, at least in some cases, which could also help attain correct diagnosis and treatment.

## Consent

Written informed consent was obtained from the patient for publication of this case report and any accompanying images. A copy of the written consent is available for review by the Editor-in-Chief of this journal.

## Competing interests

The authors declare that they have no competing interests.

## Authors’ contributions

YY wrote the manuscript. HS, HH and XY participated in the clinical management of our patient. XZ was involved in the final editing of the manuscript. All authors approved the final manuscript.

## References

[B1] Okines A Thomson CS Radstone CR Second primary malignancies after treatment for malignant lymphoma Br J Cancer 2005 93 418 424 10.1038/sj.bjc.6602731 16106249 PMC2361580

[B2] Mudie NY Swerdlow AJ Higgins CD Risk of second malignancy after non-Hodgkin's lymphoma: a British Cohort Study J Clin Oncol 2006 24 1568 1574 10.1200/JCO.2005.04.2200 16520465

[B3] Moser EC Noordijk EM van Leeuwen FE Risk of second cancer after treatment of aggressive non-Hodgkin's lymphoma; an EORTC cohort study Haematologica 2006 91 1481 1488 17043014

[B4] Okawa Y Shimada T Nagasaki E [Pulmonary cryptococcosis occurring 6 months after cladribine therapy for relapsed follicular lymphoma] Rinsho Ketsueki 2006 47 650 655 16910576

[B5] Ahn IS Kim HG Ryu JS A case of pulmonary cryptococcosis with non-small cell lung cancer in idiopathic CD4+ T-lymphocytopenia Yonsei Med J 2005 46 173 176 10.3349/ymj.2005.46.1.173 15744824 PMC2823047

